# Effects of replacing *Leymus chinensis* with whole-crop wheat hay on Holstein bull apparent digestibility, plasma parameters, rumen fermentation, and microbiota

**DOI:** 10.1038/s41598-017-02258-2

**Published:** 2017-05-18

**Authors:** Wenjing Niu, Yang He, Chuanqi Xia, Muhammad Aziz Ur Rahman, Qinghua Qiu, Taoqi Shao, Yixun Liang, Linbao Ji, Haibo Wang, Binghai Cao

**Affiliations:** 1 State Key Laboratory of Animal Nutrition, College of Animal Science and Technology, China Agricultural University, Beijing, China; 2 Gomal College of Veterinary Sciences, Gomal University, D.I. Khan, KPK Pakistan

## Abstract

Twelve Holstein bulls were used in a 4 × 4 Latin square design to investigate the effects of using whole-crop wheat hay (WCWH) as a substitute for *Leymus chinensis* (LC) on apparent digestibility, plasma parameters, ruminal fermentation, and microbial communities. Experimental treatments were four proportions of WCWH, 0, 33, 67, and 100%, as a substitute for LC (WCWH0, WCWH33, WCWH67, and WCWH100, respectively). The WCWH100 group showed a higher nutritional intake of crude protein (CP) and higher apparent digestibility of organic matter (OM), CP, and ether extract (EE) than the WCWH0 group (*P* < 0.05). Urea N, NH3-N, isobutyrate and isovalerate levels were higher (*P* < 0.05) in the WCWH100 group than in the WCWH0 group. 16S rRNA high-throughput sequencing analysis revealed similarities in the community composition, species diversity and relative abundance of dominant bacteria at the phylum and genus levels among the four groups. Collectively, our data indicated that WCWH can be used to replace LC in the diet of finishing dairy bulls without having a negative impact on apparent digestibility, plasma parameters, and ruminal bacteria composition. These results offer the first deep insight into the effects of replacing LC with WCWH on the performance parameters and rumen microbiota in Holstein bulls, and may aid in ruminant farming.

## Introduction

*Leymus chinensis* (LC), which is widely distributed throughout the Eurasian Steppe, including the Songnen Plain and the eastern Inner Mongolian Plateau in China, is a perennial species of Gramineae^1^. Although LC is considered a kind of middle-quality roughage, livestock farms must purchase LC, involving thousands of kilometres of shipping transportation, thereby contributing to a large amount of additional expense. Potential alternative roughage sources must be found due to the high long-distance transportation expenses of LC and the small profit margin of the ruminant industry.

Wheat is one of the most important crops and wheat straw is the second largest biomass feedstock in the world^2^. However, there are more than 110 million tons of wheat straw every year in China, and most of them cannot be fully used^3^. Burning wheat straw results in severe resource waste and air pollution^4^. Many studies have been conducted to attempt to make full use of wheat straw. Both Owens *et al*.^5^ and Keady *et al*.^6^ found that feeding fermented whole-crop wheat (WCW) to beef cattle increased dry matter intake (DMI) and improved rumen fermentation. Weinbery *et al*.^7^ suggested that prolonged storage might decrease the dry matter (DM) and neutral detergent fibre (NDF) digestibility values. In addition, Geough *et al*.^8^ found that increasing the grain to straw + chaff ratios of WCW silage in the diets of finishing beef steers can serve as an effective method for enteric methane abatement. However, little information can be found about the effects of whole-crop wheat hay (WCWH) on apparent digestibility, plasma parameters and rumen fermentation of Holstein bulls. Therefore, using WCWH in place of LC is of high interest and an immediate necessity. Furthermore, replacing LC with WCWH can save energy, reduce pollution, and promote the integration of agriculture and animal husbandry.

The rumen is inhabited by diverse microbial symbionts, which play a major role in digesting and transforming a fraction of the feed into microbial proteins and volatile fatty acids^9^. The ruminal bacteria are closely associated with the feed, and different forages strongly influence the rumen metabolism, thus affecting the rumen microbiota^10^. For example, using 16S rRNA gene-cloning library technology, Kong *et al*.^11^ found different species in the bacterial communities of cows fed alfalfa or triticale diets. Yuhong *et al*.^12^ revealed that partial replacement of corn grain and cotton seed meal with ensiled mulberry leaves and sun-dried mulberry fruit pomace did not significantly affect the composition of the ruminal microflora. Thus, it is necessary to explore the changes in the rumen microbial community composition when the bulls are fed WCWH as a replacement for LC. To determine the most appropriate replacement level, multiple levels of LC replacement with WCWH were evaluated in this study. We hypothesize that WCWH can replace LC in the diet of finishing dairy bulls without negative effects. The purpose of this research was to determine the effects of WCWH as a substitute for LC on apparent digestibility, plasma parameters, rumen fermentation, and bacterial communities in Holstein bulls.

## Results

### Nutrient intake and apparent digestibility

The results of feed intake and apparent digestibility are listed in Table 1. The DM intake of bulls fed WCWH0 was significantly higher than that of bulls fed WCWH33 (*P* < 0.05), but was not different from that in the other groups. The OM intake of bulls fed WCWH0 was higher than that of bulls fed WCWH33 or WCWH100 (*P* < 0.05). However, bulls fed WCWH67 or WCWH100 had a higher CP intake (*P* < 0.001), and a lower NDF and acid detergent fibre (ADF) intake (*P* < 0.01) than bulls fed WCWH0. The EE intake was not significantly affected by the treatments (*P* > 0.05). DM, NDF and ADF apparent digestibility did not differ significantly among the dietary groups (*P* > 0.05). However, bulls fed WCWH100 had a significantly higher apparent digestibility of OM, CP and EE (*P* < 0.01) than bulls fed WCWH0.

**Table 1:** The effects of treatments on feed intake and apparent digestibility of Holstein dairy bulls during the fattening period^1,2^.

Item	Dietary treatment^1^					
	WCWH0	WCWH33	WCWH67	WCWH100	SEM^3^	*P*-value
Nutrient intake, kg/d						
DM	13.5^a^	12.6^b^	13.2^ab^	12.9^ab^	0.48	0.047
OM	12.7^a^	11.7^b^	12.2^ab^	11.8^b^	0.48	0.020
CP	1.51^b^	1.47^b^	1.65^a^	1.71^a^	0.06	<0.001
EE	0.25	0.24	0.25	0.25	0.01	0.723
NDF	5.23^a^	4.81^b^	4.64^bc^	4.38^c^	0.21	<0.001
ADF	2.74^a^	2.61^ab^	2.53^b^	2.33^c^	0.12	<0.001
Apparent digestibility of nutrients, %						
DM	76.9	74.2	77.6	78.5	2.90	0.191
OM	79.6^bc^	77.4^c^	81.4^ab^	82.2^a^	2.42	<0.001
CP	74.2^bc^	71.6^c^	77.3^ab^	81.0^a^	2.89	<0.001
EE	66.7^b^	66.2^b^	68.8^b^	76.3^a^	4.30	0.007
NDF	66.7	63.1	67.5	69.7	4.11	0.170
ADF	60.4	56.4	62.6	61.6	4.86	0.297

^1^WCWH0 = 0% of *Leymus chinensis* was replaced by whole-crop wheat hay; WCWH33 = 33% of *Leymus chinensis* was replaced by whole-crop wheat hay; WCWH67 = 67% of *Leymus chinensis* was replaced by whole-crop wheat hay; WCWH100 = 100% of *Leymus chinensis* was replaced by whole-crop wheat hay.

^2^a–c: Means within the same row without the same letter superscripts are significantly different (Tukey’s test; *P* < 0.05).

^3^SEM: Standard error of the mean.

### Plasma metabolites

The effects of dietary groups on plasma metabolites are shown in Table 2. The WCWH100 treatment group had a higher level of urea N (*P* < 0.01) than the other dietary groups. However, no marked differences among the treatments were detected for the plasma glucose, triglyceride, cholesterol, total protein, albumin, high-density lipoprotein cholesterol (HDL-C), low-density lipoprotein cholesterol (LDL-C), and β-hydroxybutyrate (D3HB) concentrations.

**Table 2:** The effects of dietary treatments on plasma biochemical parameters of Holstein dairy bulls during the fattening period^1,2^.

Item	Dietary treatment^1^					
	WCWH0	WCWH33	WCWH67	WCWH100	SEM^3^	*P*-value
Glucose (mmol/L)	4.22	4.38	4.40	4.47	0.22	0.454
Triglyceride (mmol/L)	0.17	0.18	0.16	0.17	0.03	0.509
Cholesterol (mmol/L)	2.56	2.73	2.69	2.71	0.28	0.806
Total protein (g/L)	62.6	64.4	63.1	64.5	4.30	0.898
Albumin (g/L)	29.8	31.4	31.0	31.7	1.67	0.417
Urea N (mmol/L)	2.58^b^	2.77^b^	2.75^b^	3.15^a^	0.22	0.007
HDL-C^4^ (mmol/L)	0.99	1.07	0.99	1.06	0.09	0.498
LDL-C^5^ (mmol/L)	0.82	0.84	0.85	0.56	0.09	0.939
D3HB^6^ (mmol/L)	0.22	0.24	0.20	0.23	0.03	0.367

^1^WCWH0 = 0% of *Leymus chinensis* was replaced by whole-crop wheat hay; WCWH33 = 33% of *Leymus chinensis* was replaced by whole-crop wheat hay; WCWH67 = 67% of *Leymus chinensis* was replaced by whole-crop wheat hay; WCWH100 = 100% of *Leymus chinensis* was replaced by whole-crop wheat hay.

^2^a–b: Means within the same row without the same letter superscripts are significantly different (Tukey’s test; *P* < 0.05).

^3^SEM: Standard error of the mean.

^4^High density lipoprotein cholesterol.

^5^Low density lipoprotein cholesterol.

^6^β-Hydroxybutyrate.

### Rumen fermentation characteristics

The parameters of rumen fermentation characteristics are presented in Table 3. The pH values of rumen liquid were similar (*P* > 0.05) among the groups. The concentrations of NH_3_-N, isobutyrate and isovalerate in the WCWH100 group were higher (*P* < 0.01) than those in the WCWH0 group. There were no differences in the concentrations of total volatile fatty acids (TVFA), acetate, propionate, butyrate or in the acetate / propionate ratio among the dietary treatments (*P* > 0.05).

**Table 3:** The effects of dietary treatments on rumen fermentation of Holstein dairy bulls during the fattening period^1,2^.

Item	Dietary treatment^1^					
	WCWH0	WCWH33	WCWH67	WCWH100	SEM^3^	*P*-value
pH	6.79	6.71	6.73	6.74	0.09	0.622
NH_3_-N	3.86^c^	4.59^bc^	6.34^a^	5.49^ab^	0.78	<0.001
TVFA^4^ (mmol/L)	82.3	86.3	91.9	90.0	10.7	0.596
VFA^5^ (mmol/L)						
Acetate	47.5	48.1	52.7	54.5	6.29	0.328
Propionate	20.0	20.4	21.7	20.8	3.70	0.925
Isobutyrate	0.32^b^	0.46^a^	0.48^a^	0.54^a^	0.08	0.002
Butyrate	12.9	15.3	15.0	12.1	2.51	0.226
Isovalerate	0.79^c^	0.88^bc^	1.16^ab^	1.26^a^	0.21	0.007
Valerate	0.78	1.20	0.91	0.82	0.26	0.108
Acetate/Propionate	2.57	2.46	2.51	2.74	0.24	0.390

^1^WCWH0 = 0% of *Leymus chinensis* was replaced by whole-crop wheat hay; WCWH33 = 33% of *Leymus chinensis* was replaced by whole-crop wheat hay; WCWH67 = 67% of *Leymus chinensis* was replaced by whole-crop wheat hay; WCWH100 = 100% of *Leymus chinensis* was replaced by whole-crop wheat hay.

^2^a–c: Means within the same row without the same letter superscripts are significantly different (Tukey’s test; *P* < 0.05).

^3^SEM: Standard error of the mean.

^4^Totalvolatile fatty acids.

^5^Volatile fatty acids.

### Rumen bacteria composition in different diet groups

**Figure 1:**
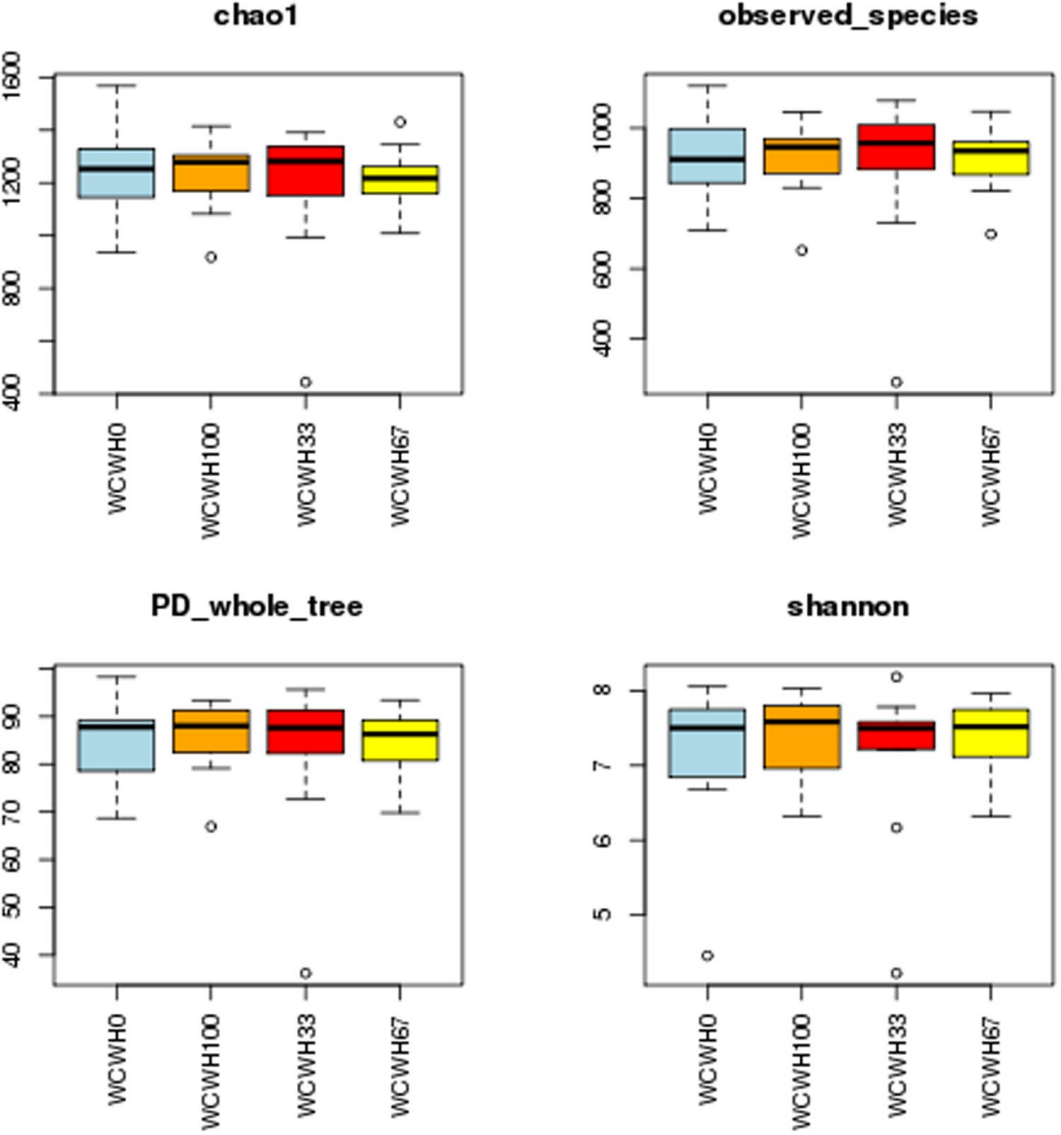
Richness estimates and diversity indices for bacteria within each diet group. The top and bottom boundaries of each box represent the 75th and 25th quartile values, respectively. The horizontal lines inside each box represent the median values.

**Figure 2:**
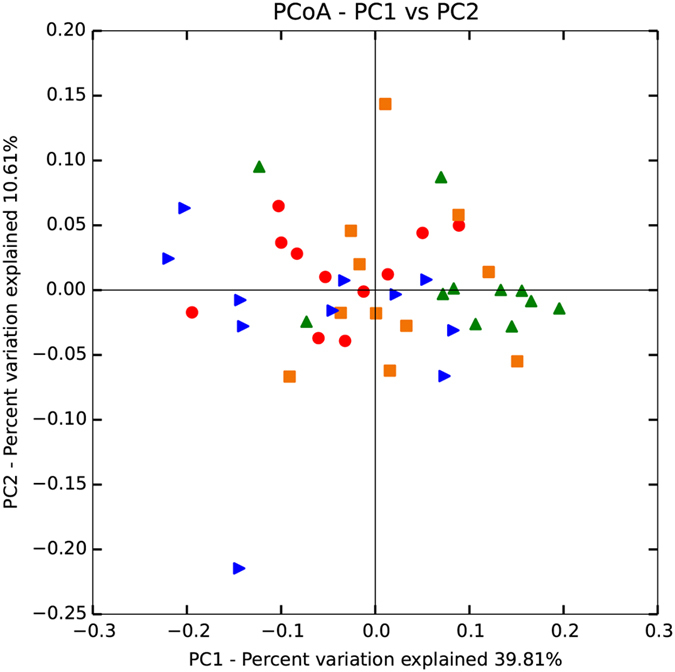
Principal Coordinate Analysis (PCoA) of rumen bacterial community structures of bulls in the four groups. The PCoA plots were constructed using the weighted UniFrac method.

A total of 1,563,811 high-quality sequences, with an average of 32,579 sequences per sample, were retained after quality control and chimaera removal. These sequences, with an average length of 420 bp, were assigned to 2091 operational taxonomic units (OTUs) of rumen bacteria based on a 97% similarity cut-off. After normalization to 13,125 reads, richness estimates and diversity indices (Fig. 1, Supplementary Table 1) were evaluated. Our results indicated that richness estimates and diversity indices in the rumen microbiota did not differ significantly (*P* > 0.05). We further analysed the beta diversity, which is shown by principal coordinate analysis (PCoA) in Fig. 2, with colours representing different diet groups. PCoA plots of bacterial 16S rRNA showed no obvious clusters among the four diet groups using PC1 and PC2 (39.81% and 10.61%, respectively, of the explained variance). Meanwhile, the PERMANOVA results, which were analysed using the original distance matrix, indicated no marked differences among the four groups (*P* = 0.451). The PERMANOVA results for pair-wise tests between the groups were presented in Supplementary Table 2.

**Figure 3:**
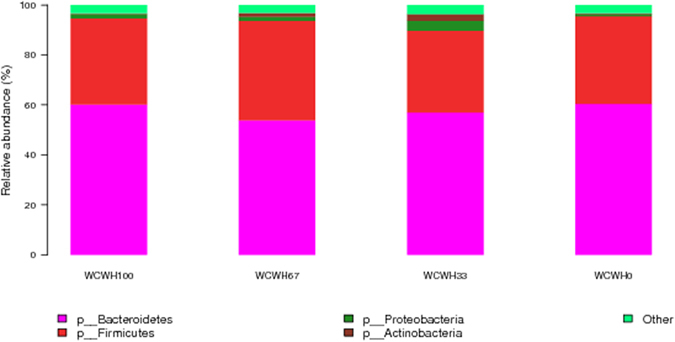
The effects of dietary treatments on the relative abundance of phyla in the ruminal bacterial community.

**Figure 4:**
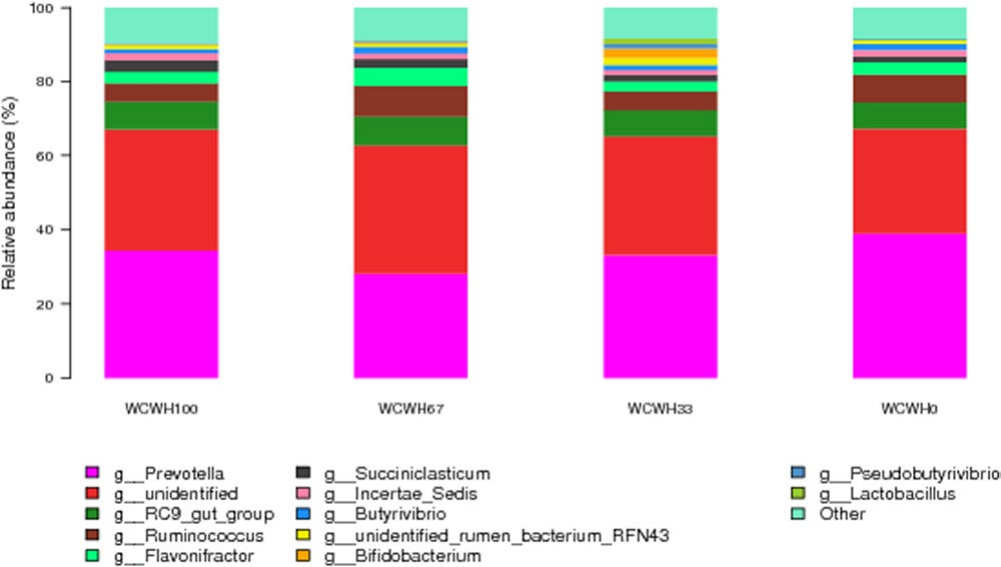
The effects of dietary treatments on the relative abundance of genera in the ruminal bacterial community.

Based on the taxonomic guidelines of the Silva project^13^, microbial compositions at the phylum and genus levels are presented in Figs 3 and 4, respectively. There were 21 identified phyla of rumen bacteria (Supplementary Table 3). In all samples, the most dominant phyla were *Bacteroidetes* (57.98%) and *Firmicutes* (35.20%), followed by *Proteobacteria* (1.96%) and *Actinobacteria* (1.35%). Minor phyla included *Fibrobacteres* (0.83%), *Spirochaetae* (0.77%), and *Lentisphaerae* (0.65%). The other known phyla occupied 1.15% of the rumen bacteria. The most abundant genera were *Prevotella* (33.86%), *RC9 gut group* (7.36%), *Ruminococcus* (6.23%), *Flavonifractor* (3.35%), and *Succiniclasticum* (2.15%). Minor genera, such as *Incertae_sedis*, *Butyrivibrio*, *unidentified rumen bacterium RFN43*, *Saccharofermentans*, *Fibrobacter*, *Treponema*, *Pseudobutyrivibrio*, *Selenomonas*, and *Acetitomaculum*, accounted for 1.61%, 1.47%, 1.33%, 0.91%, 0.83%, 0.70%, 0.67%, 0.62% and 0.55%, respectively, of the bacterial community. Other known genera accounted for 6.86% of the bacterial composition, and 31.51% of the sequences were unclassified at the genus level. Supplementary Table 4 shows the relative abundance of genera in all ruminal samples.

### Effects of dietary treatments on rumen microbial diversities and relative abundance

Replacing LC with WCWH did not affect the richness estimates and diversity indices (*P* > 0.05) (Fig. 1). The Shannon index represents species richness and evenness of the samples; Chao1 and observed species are primarily used to evaluate the richness of the microbial community.

Among all of the phyla detected, no significant differences were observed in ruminal bacteria community composition and relative abundance among the four groups (Fig. 3 and Supplementary Table 3). At the genus level, there were no significant differences among the four diet groups except for some minor genera, such as *Staphylococcus*, *Saccharopolyspora and Laceyella* (Fig. 4 and Supplementary Table 4).

### Correlation analysis

**Figure 5:**
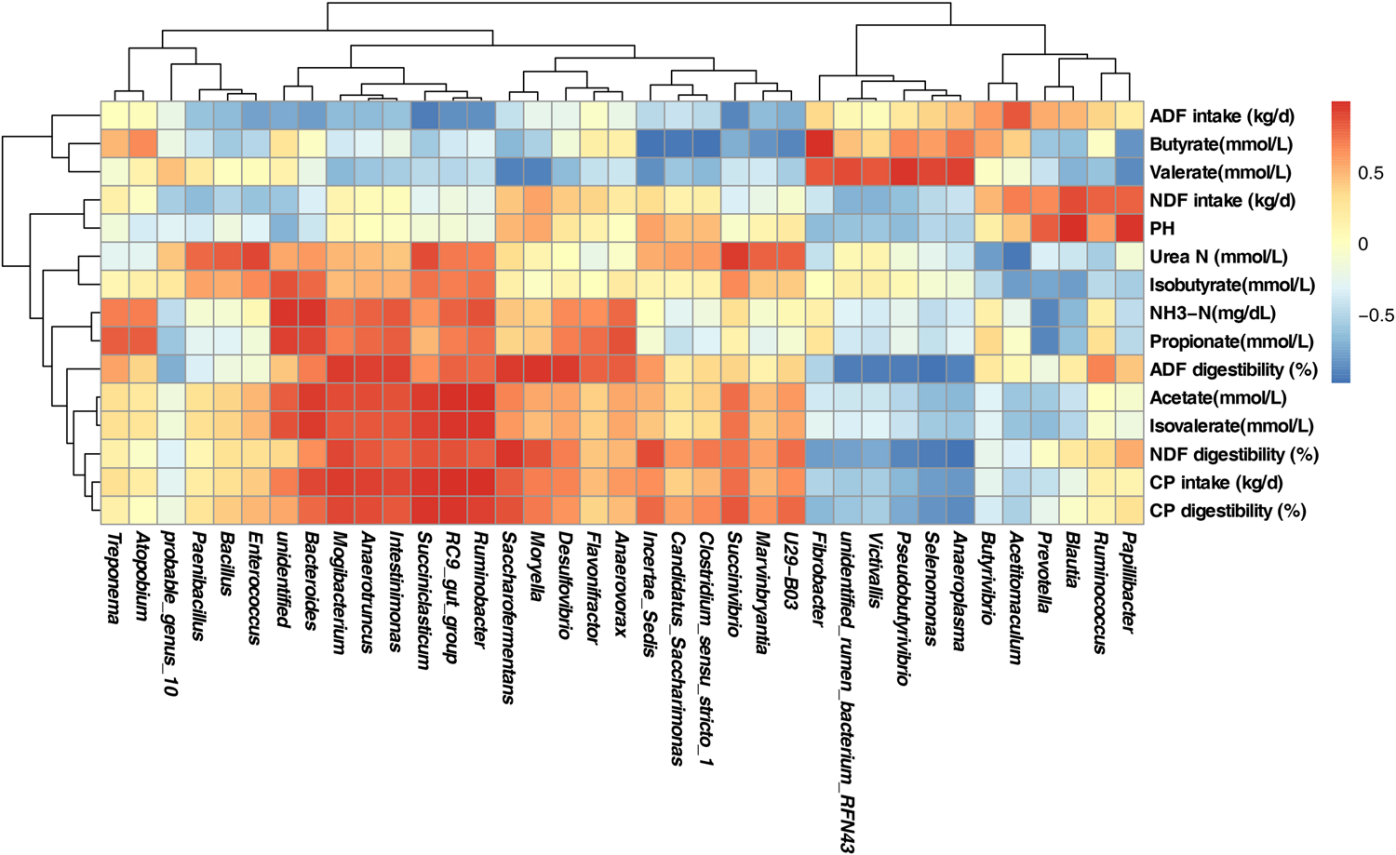
Correlation between physiological / production parameter and genus abundance. The top and left hierarchical cluster based on the corresponding correlation matrix between physiological / production parameters and genus abundance. The similar clusters were found with complete linkage method. Only the predominant bacterial genera (representing at least 1% of the bacterial community in at least one sample) are presented; Cells are coloured based on the Pearson correlation coefficient between physiological / production parameter and genus abundance. The red colour represents a positive correlation; the blue colour represents a negative correlation.

The relationships between physiological / production parameters and genus abundance (representing at least 1% of the bacterial community in at least one sample) were evaluated in this study (Fig. 5). The results showed that the CP intake correlated positively with the abundance of *RC9_gut_group* (r = 0.998; *P* = 0.002), *Succiniclasticum* (r = 0.961; *P* = 0.039) and *Ruminobacter* (r = 0.987; *P* = 0.013). The apparent digestibility of ADF correlated positively with the abundance of *Saccharofermentans* (r = 0.956; *P* = 0.044), *Moryella* (r = 0.975; *P* = 0.025), *Mogibacterium* (r = 0.951; *P* = 0.049) and *Desulfovibrio* (r = 0.952; *P* = 0.048), and negatively with the abundance of *Selenomonas* (r = −0.980; *P* = 0.020). The apparent digestibility of NDF correlated positively with the abundance of *Saccharofermentans* (r = 0.968; *P* = 0.032), and negatively with the abundance of *Anaeroplasma* (r = −0.979; *P* = 0.021). The apparent digestibility of CP correlated positively with the abundance of *RC9_gut_group* (r = 0.961; *P* = 0.039) and *Succiniclasticum* (r = 0.975; *P* = 0.025). The concentration of urea N correlated negatively with the abundance of *Acetitomaculum* (r = −0.953; *P* = 0.047). The ruminal pH correlated positively with the abundance of *Papillibacter* (r = 0.959; *P* = 0.041) and *Blautia* (r = 0.982; *P* = 0.018). The concentration of NH_3_-N correlated positively with the abundance of the unidentified genus belonged to the phylum *Firmicutes* (r = 0.978; *P* = 0.022), *Bacteroides* (r = 0.968; *P* = 0.032), The concentration of acetate correlated positively with the abundance of *RC9_gut_group* (r = 0.983; *P* = 0.017), *Succiniclasticum* (r = 0.956; *P* = 0.044), *Ruminobacter* (r = 0.987; *P* = 0.013). The concentration of butyrate correlated positively with the abundance of *Fibrobacter* (r = 0.995; *P* = 0.005), and negatively with the abundance of *Incertae_Sedis* (r = −0.967; *P* = 0.033), *Candidatus_Saccharimonas* (r = −0.961; *P* = 0.039), and *Clostridium_sensu_stricto_1* (r = −0.982; *P* = 0.018). The concentration of isovalerate correlated positively with the abundance of *RC9_gut_group* (r = 0.964; *P* = 0.036), *Ruminobacter* (r = 0.976; *P* = 0.024), and *Bacteroides* (r = 0.954; *P* = 0.046).

## Discussion

Feed intake can be affected by both physiological factors and external factors, including dietary chemical composition, environmental conditions, and animal learning abilities^14, 15^. Regarding chemical components, DMI generally decreases with increasing NDF concentration^16^. In the present study, the WCWH0 diet had a higher NDF content but had a significantly higher DMI. This maybe because the WCWH0 diet had a numerically higher DM digestion rate and faster flow from the reticulo-rumen^17^. Additionally, the greater intake of OM, NDF, and ADF in the WCWH0 group may have been related to greater OM, NDF, and ADF content in the WCWH0 diet (Table 4)^18^, reflecting the chemical composition of the diet consumed in the case of the similar DMI. Steen *et al*.^19^ reported that digestibility was a crucial factor in regulating forage intake. However, there were lower intake of OM, NDF and ADF but higher OM digestibility and similar digestibility of NDF and ADF in the WCWH100 diet group. These findings were consistent with previous reports^20^, indicating that digestibility was not the dominant factor controlling forage intake. The significantly higher CP intake of bulls fed the WCWH100 diet compared with the WCWH0 diet might be caused by bulls favouring the WCWH100 diet and ingesting more.

**Table 4:** Ingredients and nutrient compositions of the dietary treatments.

Item	Dietary Treatments^1^			
	WCWH0	WCWH33	WCWH67	WCWH100
Ingredient, % (DM basis)				
corn grain	22.3	24.1	25.9	27.6
wheat grain	26.5	23.8	21.1	18.5
soybean meal	3.45	2.31	1.14	0.00
rapeseed meal	0.70	1.54	2.39	3.23
wheat bran	0.00	1.14	2.32	3.47
NaHCO3	0.55	0.55	0.55	0.55
NaCl	0.55	0.55	0.55	0.55
limestone	0.44	0.48	0.51	0.55
mineral-vitamin premix^2^	0.55	0.55	0.55	0.55
WCWH	0.00	14.9	30.2	45.0
CWR	45.0	30.2	14.9	0.00
Chemical composition^3^, % (DM basis)				
OM	94.0	92.8	91.8	91.1
CP	12.1	12.2	12.2	12.1
EE	1.02	1.02	1.02	1.02
NDF	31.9	29.9	27.9	25.9
ADF	17.5	16.6	15.7	14.9

^1^WCWH0 = 0% of *Leymus chinensis* was replaced by whole-crop wheat hay; WCWH33 = 33% of *Leymus chinensis* was replaced by whole-crop wheat hay; WCWH67 = 67% of *Leymus chinensis* was replaced by whole-crop wheat hay; WCWH100 = 100% of *Leymus chinensis* was replaced by whole-crop wheat hay.

^2^Every kilogram of mineral–vitamin premix contained the following: 3500 IU Vitamin A, 3000 IU Vitamin D_3_, 45 IU Vitamin E, 63 mg Zn, 60 mg Fe, 98.6 mg Mn, 20 mg Cu, 1.1 mg I, 0.50 mg Se, and 0.45 mg Co.

^3^OM = organic matter; CP = crude protein; NDF = neutral detergent fibre; ADF = acid detergent fibre.

In this study, DM apparent digestibility had no significant differences among the treatments because the diets could have remained in the rumen for the similar length of time and had similar enzymatic activity, resulting in similar DM digestibility^21^. The higher apparent digestibility of OM and EE in the WCWH100 diet group than in the WCWH0 diet group was expected. These differences could be attributed to the high starch content of the WCWH100 diet, which was easily digested and absorbed by animals^20^. The higher CP digestibility in the WCWH100 group was expected and can be explained by the faster and more extensive CP degradation of wheat grain in the rumen^22^. Walsh, K. *et al*.^23^ observed that lower rumen pH values selectively restricted the activity of cellulolytic bacteria thereby reducing the fibre digestibility. Therefore, the undifferentiated digestibility of NDF and ADF among dietary treatments could be attributed to the normal and similar rumen pH values generating similar levels of activity of cellulolytic bacteria.

In the present study, the concentrations of plasma glucose and triglyceride were similar among the dietary treatments potentially due to the similar DM intake and ruminal propionate concentration. Jolazadeh *et al*.^24^ reported that similar DM intake had no effect on Holstein bulls plasma glucose and triglyceride concentrations. Moreover, propionate from rumen fermentation is the major substrate for hepatic gluconeogenesis in ruminants^23, 25^. Plasma cholesterol, HDL, and LDL were not affected by the diets and showed no hepatic or cardiac impairment in bulls^26^. The absence of effects of dietary treatments on the plasma albumin and total protein concentrations possibly resulted from the similar flow of microbial protein to the intestine^27^ and the amount of amino acids available for absorption^28^.

The plasma urea N was produced by protein hydrolysis and amino acid metabolism, and its concentration depends on the level of crude protein^26^. In the current study, the WCWH100 group had a greater plasma urea concentration than the other groups, which could be attributed to the relatively higher protein intake of WCWH^29, 30^. Butyrate, which comes from rumen fermentation, is mostly converted to D3HB through the rumen epithelium^31^. Therefore, the undifferentiated plasma D3HB concentrations among treatment diets resulted from similar butyrate concentrations in the rumen during fermentation, which is consistent with the findings of Thorp *et al*.^32^.

In this study, rumen pH values were in the normal 6.4–6.8 range^33^, indicating that the rumen internal environment was static to some extent when WCWH was substituted for LC. Rumen NH_3_-N is the degradation product of feed protein^34^. The greater NH_3_-N concentrations in the WCWH100 group resulted from the relatively higher protein intake and digestibility in the WCWH100 treatment. Fibre-degrading ruminal bacteria utilize ammonia as their primary source of N for growth^35^. Therefore, increasing the ammonia level improves fibre digestion in the rumen. The total VFA concentrations were unaffected among the groups, which might be the result of the same ratio of forage to concentration of treatment diets^36^, or might be caused by the fact that the output of total VFA was equivalent to the amount of absorption in the rumen among all treatments^37^. In the present research, the observed concentrations of acetate and propionate, and the acetate to propionate ratio were not significantly different, probably because of the similar ruminal pH values caused by the dietary-associated shifts^38^.

Isobutyrate and isovalerate levels significantly increased with increasing proportions of WCWH, which were due to the increased metabolism caused by the WCWH diets^39^. Consistent with our report, branched-chain VFAs (BCVFAs) are particularly required by most fibre-degrading bacteria and can increase fibre degradation^40^. In response to WCWH100, interesting links exist among BCVFA, NH_3_-N, OM and CP digestibility. The increased ruminal NH_3_-N concentrations suggested that the increased OM digestibility promoted the capacity of the rumen microbes to utilize the NPN (Non-Protein Nitrogen) supplied in the diets^41^. In addition, the increased ammonia concentrations in bulls fed the WCWH100 diet may indicate a declined utilization efficiency of NH_3_-N by the rumen microorganisms^42^, resulting from an increase in the proportion of BCVFAs observed in the WCWH100 group^43^. BCVFAs are mainly end products of protein fermentation and are, along with ammonia, often used as quick indicators of protein fermentation in the rumen^44^. Thus, the higher concentration of BCVFAs was also associated with increases in protein digestibility and ammonia concentration.

In the present study, Good’s coverage estimate was 0.98, indicating that the research recovered 98% of all OTUs computed at a 0.97 similarity. The similar richness estimates and diversity indices indicated that the rumen microbial diversity was similar among the four diet groups^45^. In general, diet is considered the most important determining factor of microbial composition^46^. In our study, the four groups had the same concentrate to forage ratio, as well as the same levels of protein and energy, and the bulls were raised under the same ambient conditions, which might account for the similarities in rumen microbial diversity. In addition, the PCoA examining the phylogenetic divergence among the OTUs did not significantly cluster, further indicating the similarities of microbial communities in different treatment groups. The rumen microbial community is a complex network and ferments the roughage ingested by the ruminant. There is a dependence among host physiology, diet composition, and microbial richness^47–49^. The dominant microbes must take the responsibility for a large amount of the transformation of ingested forage, especially of protein, cellulose, starch, and so on, because these are the principal energy-yielding substrates that are used for the growth of microorganisms^50^. In this study, the most abundant phyla were *Bacteroidetes*, *Firmicutes*, and *Proteobacteria*, which are the main bacterial phyla and play important roles in rumen fermentation^51–53^. Based on the data set presented in the current study, *Bacteroidetes* was more predominant than *Firmicutes*, consistent with the result of Jami and Mizrahi^54^, who reported that the rumen pH was approximately 6.51 and the *Bacteroidetes* proportion (50%) was higher than that of *Firmicutes* (43%). However, the lower rumen pH may result in a substantially decreased proportion of *Bacteroidetes* and an increased proportion of *Firmicutes* in the rumen microbial community^55^. Therefore, these studies indicated that the phylum *Bacteroidetes* maybe predominant instead of *Firmicutes* in the case of the normal pH range caused by our experimental diets. In our study, the *Proteobacteria* represented 1.96% of total bacteria in rumen. In general, the phylum *Proteobacteria* is dominant in the neonatal stage, followed by a sudden and sharp decline in its proportion, with *Proteobacteria* reaching the lowest proportion while that of *Bacteroidetes* becomes the highest^56^.

Additionally, *Bacteroidales* are capable of degrading cellulose, and their genomes encode degradable ability of plant polysaccharide^57, 58^. Therefore, the lack of differences in *Bacteroidales* abundance might explain the similar ADF digestibility in the current study. Within *Bacteroidetes*, the most dominant genus was *Prevotella* in the rumen, accounting for 47% of the total bacterial sequences. For ruminants, *Prevotella* owns the dipeptidyl peptidase type IV activity of rate-limiting, which is responsible for cleaving oligopeptides. Therefore, it plays an important role in protein metabolism, especially in breaking down oligopeptides in the rumen^59^. The different treatments did not affect the relative abundance of *Prevotella*, similar to the findings of a previous study^60^. *Prevotella* is a predominant organism in bulls fed both forage and grain^48^ and contributes to the majority of hereditary and metabolic variety of the microflora^61^.

Within the phylum *Firmicutes*, *Ruminococcus* is a predominant genus that tended to decrease numerically with increasing WCWH substitution levels. *Ruminococcus* contains two kinds of powerful fibre-degrading bacteria, *Ruminobacter albums* and *Ruminococcus flavefaciens*, which can produce large amounts of cellulase and hemicellulase to decompose plant fibre^62^. The fibre content decreased with the increasing WCWH substitution levels, and the proportion of *Ruminococcus* in the bacterial community decreased because of the lesser amount of substrate fibre available for them. The genus *Succiniclasticum*, which represents up to 2.15% of the total bacterial sequences, is involved in fermenting succinate and transforming it into propionate^63^. Additionally, the proportion of *Succiniclasticum* among the total sequences tended to increase numerically with increasing WCWH substitution level, which may indicate resource competition among the rumen bacteria^64^. This finding can be attributed to the ability of *Succiniclasticum* to decompose starch in the rumen of cattle^49^. In the present research, *Succiniclasticum* can lead to a trend of decrease in NDF digestibility with an increase in NDF content when diets have the same levels of protein and energy. In addition, this study found *Butyrivibrio* (belonging to *Firmicutes*) at 1.47% among the total bacteria in the rumen of Holstein bulls. The species of *Butyrivibrio*, which can produce mucosal butyrate and release butyrate close to the epithelium, may improve the bioavailability of butyrate for the host^65^.

*Fibrobacteres* species are main cellulolytic bacteria in the rumen^45^. In our results, the sequences of *Fibrobacteres* accounted for an average of only 0.83% of the total bacterial community, which was similar to a previous study by Zened *et al*.^45^. The minor and indifferent abundance of *Fibrobacteres* might account for the lack of change in NDF and ADF digestibility. Within the phylum *Spirochaetae*, the genus *Treponema*, which commonly exists in the rumen, is involved in degrading soluble fibre. Compared with the WCWH0 group, the other groups had not different abundance of *Treponema*, potentially because of similar pectin concentrations among the four treatments^12^.

In the present study, correlation analysis revealed that there was a relationship between nutrition intake /digestibility and rumen microbial proportion. In particular, the increased intake and apparent digestibility of CP were associated with the genera *RC9_gut_group* and *Succiniclasticum*, which belong to the phyla *Bacteroidetes* and *Firmicutes*, respectively. As reported previously, the nitrogen source in protein is essential to the proliferation of *RC9_gut_group* ^66^. Additionally, we concluded that the *Succiniclasticum* may play a role in the degradation of protein. In addition, both plasma metabolites and rumen metabolites were relevant to microorganisms. For instance, the correlation analysis showed that the concentrations of NH_3_-N and isovalerate were linked to enrichments in *Bacteroides*, which has been postulated to be able to degrade cellulose^58^. Isovalerate can be utilized by fibre-degrading ruminal bacteria and may be related to the increased ammonia concentration^40, 44^. In general, further studies are needed to evaluate the relationships between physiological parameters and other rumen microbes, such as protozoa, archaea, and fungi in the complex system of rumen microorganisms.

There has been no previous research examining the effects of WCWH as a substitute for LC on apparent digestibility, plasma parameters, rumen fermentation and especially the rumen microbiota of Holstein dairy bulls as outlined in our study. In conclusion, based on 16S rRNA high-throughput sequencing of ruminal microbiota, this study showed a detailed account of bacteria in the rumen and their relative abundances under the influence of the tested diets. The study revealed that using WCWH as a substitute for LC did not negatively affect apparent digestibility, plasma parameters, and the ruminal bacteria composition. However, as with many previous studies^12, 45, 56^, limiting the study to collect rumen fluid may result in underrepresentation of fibre-degrading microflora. Thus, we will collect both rumen fluid and solid to study microbiota in future research. The rumen microflora is a complex ecosystem that degrades forage for ruminants. It is necessary to further study whether other rumen microbes, such as protozoa, archaea, and fungi, also affect rumen characteristics.

## Materials and Methods

### Ethics Statement

Our experimental procedures were approved by the Animal Welfare and Ethics Committee of China Agricultural University (Permit No. DK1008). All experiments were performed according to the approved Regulations for the Administration of Affairs Concerning Experimental Animals (The State Science and Technology Commission of P. R. China, 1988).

### Experimental design, animal management and diet

The feeding trial was carried out at the Xinzhicheng Dairy Farm (Zhuozhou City, Hebei Province, China). In this study, 12 Holstein dairy bulls during the fattening period (body weight = 485.0 ± 40.8 kg) were allocated to one of four dietary groups in a 4 × 4 Latin square design. Each period consisted of a 17-d adaptation period and a 5-d sampling period. All of the bulls were inoculated with vaccines, de-wormed, weighed, and marked with numbered identification tags before the start of the experiment.

The WCW was harvested at the milky ripe stage and at a stubble height of 10 to 15 cm in Zhuozhou City, Hebei Province, China. Subsequently, wheat was dried in the sun and stored for animal feed. The LC was obtained via long-distance transport from Northeast China. Experimental treatments were composed of four dietary levels of WCWH, 0, 33, 67, and 100%, as a substitute proportion of LC. The diets consisted of 55% concentrate and 44% roughage, were formulated to be isonitrogenous and isocaloric, and met the nutritional requirements of beef cattle^67^. Chemical constituents and ingredients of the trial diets are shown in Table 4. Bulls were fed twice daily at 7:00 and 17:00, allowing 5 to 10% orts, and fresh drinking water was provided ad libitum.

### Sample Collection

During d18-d22 of each period, the roughage, concentrate, and orts were collected and weighed. Faecal samples were collected from the rectum daily at 6:00, 12:00, 18:00, and 24:00 from d19-d21. The feed, orts, and faecal samples were dried at 65 °C and smashed using a mill (Wiley, A. H. Thomas Co., Philadelphia, PA, USA) with a 1-mm screen, and then stored at −20 °C for further analysis of CP, DM, OM, NDF, ADF, and acid-insoluble ash (AIA).

On d18 of each period, blood (approximately 10 mL) was collected via the jugular vein into tubes containing Na-heparin before the morning feeding. Samples were centrifuged at 3,000 × *g* for 20 min at 4 °C to collect plasma, separated into three aliquots, and frozen at −20 °C for subsequent biochemical index analyses.

Approximately 100 mL of ruminal sample consisting of a mixture of liquids and solids was obtained from the oesophageal tube 2 h after morning feeding on day 22. The pH was immediately determined using a portable pH metre (HJ-90B, Aerospace Computer Company, Beijing, China). Next, 0.25 mL of metaphosphoric acid (25 g / 100 mL) was added to four aliquots of 1 ml rumen fluid, which were centrifuged at 15,000 × *g* at 4 °C for 15 min to determine VFA and NH_3_–N concentrations. Three aliquots of 1-mL samples were taken and kept in liquid N for rumen bacterial 16S rRNA analysis.

### Chemical analysis

The EE, DM, and ash of feeds, refusal, and faecal samples were measured by methods No. 920.39, 934.01 and 924.05 of the Association of Official Analytical Chemists (AOAC 1990)^68^, respectively. The OM content was calculated using the following formula: 100- the percentage of ash. ADF and NDF were examined using the method described by Vansoest *et al*.^69^ using the Ankom Fibre Analyser (Ankom Technology, Fairport, NY). CP was measured according to the Kjeldahl method (AOAC, 1990; method 990.03).

Apparent digestibility in the digestive tract was calculated using the endogenous tracer AIA. The AIA values of the diets, orts, and faeces were analysed following the method described by Van Keulen and Young^70^, with the formula depicted by Zhong *et al*.^71^. The formula is as follows: D = [1 − (Ad × Nf) / (Af × Nd)] × 100, where Ad (g / kg) and Af (g / kg) represent the AIA of the diet and faeces, respectively, and Nd (g / kg) and Nf (g / kg) represent the nutrient contents of the diet and faeces, respectively.

The plasma biochemical indicators were determined using an automated biochemistry analyser (Hitachi 7020; Hitachi Co., Tokyo, Japan). β-Hydroxybutyric acid, glucose, triglyceride, total protein, and plasma urea nitrogen concentrations were determined using commercial test kits (Beijing Jiuqiang Bio-Technique Co., Beijing, China) with hydroxybutyric acid dehydrogenase, glucose oxidase, glycerophosphate oxidase, biuret, and urease, respectively. The levels of total cholesterol, albumin, HDL-C, and LDL-C in plasma were determined using an enzyme method, a bromocresol green method, and a direct measurement method, respectively.

The rumen liquid NH_3_–N was measured according to Bremner and Keeney^72^ using a spectrophotometer (UV-1700, Shimadzu Corporation, Kyoto, Japan). The VFA was quantified using a high-performance gas chromatograph (HPGC; GC-2014; Shimadzu Corporation) that was equipped with a hydrogen flame detector and a capillary column (Agilent, Technologies, Wilmington, DE, USA; 30 m long, 0.32 mm diameter, 0.50 µm film).

### DNA extraction and 16S rRNA pyrosequencing

First, a 1.5 mL sample of rumen fluid was centrifuged at 1,000 × g for 10 min to discard sediment, and then the clear supernatant extract was removed by centrifugation at 12,000 × g for 10 min. Subsequently, the DNA of homogenized rumen fluid was extracted using a bacterial DNA Kit (Omega Bio-Tek, Norcross, GA, USA) according to the manufacturer’s protocol. DNA concentration and purity were evaluated by using a spectrophotometer (UV-1700, Shimadzu Corporation). Bacterial 16S rRNA genes of the V3-V4 region were amplified from extracted DNA using the barcoded primers 338 F (5′-ACTCCTACGGGAGGCAGCAG-3′) and 806R (5′-GGACTACHVGGGTWTCTAAT-3′). Each 50 μL PCR mixture consisted of a DNA sample (30 ng), a forward primer (2 μL; 10.0 μmol/L), a reverse primer (2 μL; 10.0 μmol / L), DNA template (4 μL; 2.5 μmol / L), sterile distilled water (36.7 μL), 10 × Pyrobest Buffer (5 μL), and Pyrobest DNA Polymerase (0.3 μL; 2.5 U / μL, TaKaRa Code: DR005A). The amplicon mixture was applied to the MiSeq Genome Sequencer (Illumina, San Diego, CA, USA) under the following conditions: 95 °C for 5 min, followed by 25 cycles of 95 °C for 30 s, 56 °C for 30 s, and 72 °C for 40 s, along with an extension at 72 °C for 10 min and storage at 4 °C. Three PCR replicates were performed for each sample. These PCR products were pooled, purified, and then quantified using a blue fluorescence quantitative system (Quanti Fluor^TM^-ST, Promega Corporation, Madison, WI, USA). Finally, high-throughput sequencing was performed using the Illumina MiSeq platform (San Diego, CA, USA) according to the manufacturer’s instructions.

### Sequence analyses

The sequences were analysed using the Quantitative Insights Into Microbial Ecology (QIIME) V1.8 pipeline^73^. Low-quality reads, which were selected based on sequence length, quality, primers, and tags, were removed in QIIME V1.8. The Uchime algorithm^74^ carried out in Usearch soft ware was applied to remove chimeric sequences. Short tags were removed using Mothur V.1.33.3^75^. The cluster command in Mothur was used to classify the clean and high-quality sequences as bacteria. Downstream analysis (richness estimates, diversity indices and beta diversity) for OTU classification was conducted for bacteria community comparisons. The OTUs were defined by Uclust^76^ with a 97% similarity cut-off. To obtain corresponding species classification information of each OTU, the Ribosomal Database Project (RDP) classifier algorithm^77^ was used for comparative analysis of the representative OTU sequences, and all species information at various levels (phylum, genus) were annotated. Richness estimates and diversity indices including Chao 1, observed species, Good’s coverage, phylogenetic diversity whole tree (PD whole tree), and Shannon’s index were calculated using the QIIME V1.8 pipeline^73^. A PCoA^78^ based on the weighted UniFrac distances was conducted to compare all samples, and a distance-based matrices analysis (PERMANOVA) was performed to evaluate differences among samples. The vegan package in the QIIME software was used for performing PERMANOVA.

### Statistical analysis

All data were statistically analysed using PROC MIXED in SAS 9.0 (SAS Institute Inc., Cary, NC, USA) according to the following model: Yijk = μ + Pi + Tj + Ck + eijk, where Yijk is the dependent variable, μ is the general mean, Pi is the fixed effect of period (i = 1, 2, 3, or 4), Tj is the fixed effect of treatment (i = 1, 2, 3, or 4), Ck is the fixed effect of cattle and eijk is the residual effect. Differences of means in different treatments were tested using Duncan’s multiple range test and considered statistically significant at *P* < 0.05.

Correlation analysis was performed between plasma metabolites, nutrient intake / digestibility, rumen metabolites, and microbial proportion by Pearson’s correlation test using R software^79^. The top and left hierarchical cluster based on the corresponding correlation matrix between physiological / production parameters and genus abundance. The similar clusters were found with complete linkage method. Significance was declared at *P* < 0.05.

## Electronic supplementary material

α
