# Supplementary material for: Effects of replacing Leymus chinensis with whole-crop wheat hay on Holstein bull apparent digestibility, plasma parameters, rumen fermentation, and microbiota
Source: Sci Rep. 2017 May 18;7:2114. doi: 10.1038/s41598-017-02258-2 (PMC5437022; doi:10.1038/s41598-017-02258-2)
Supplement: Supplementary file 1 — α [file 41598_2017_2258_MOESM1_ESM.doc]

**Effects of replacing** ***Leymus chinensis* with whole-crop wheat hay on Holstein bull apparent digestibility, plasma parameters, rumen fermentation, and microbiota**

Wenjing Niu^1^,Yang He^1^, Chuanqi Xia^1^, Muhammad Aziz Ur Rahman^1,2^, Qinghua Qiu^1^, Taoqi Shao^1^, Yixun Liang^1^, Linbao Ji^1^, Haibo Wang^1^, Binghai Cao^1*^

Supplementary Table 1. Richness estimates and diversity indices in ruminal liquid samples from each dietary group^1^

|  |  | Dietary treatment^1^ | |  |  |  |
| --- | --- | --- | --- | --- | --- | --- |
| Item | WCWH0 | WCWH33 | WCWH67 | WCWH100 | SEM^2^ | P-value |
| chao1 | 1251 | 1243 | 1221 | 1205 | 104 | 0.918 |
| goods_coverage | 0.98 | 0.98 | 0.98 | 0.98 | 0.00 | 0.815 |
| observed_species | 929 | 947 | 913 | 903 | 79 | 0.874 |
| PD whole tree^3^ | 84.8 | 86.9 | 84.9 | 85.0 | 5.7 | 0.947 |
| shannon | 7.13 | 7.41 | 7.43 | 7.41 | 0.56 | 0.852 |

^1^ WCWH0=0% of Leymus chinensis was replaced by whole crop wheat hay; WCWH33=33% of Leymus chinensis was replaced by whole crop wheat hay; WCWH67=67% of Leymus chinensis was replaced by whole crop wheat hay; WCWH100=100% of Leymus chinensis was replaced by whole crop wheat hay;

^2^SEM = Standard error of the mean;

^3^Phylogenetic Diversity whole tree.

Supplementary Table 2. The PERMANOVA results for pair-wise tests between the groups^1^.

| Method name | p-value |
| --- | --- |
| PERMANOVA(WCWH100 vs WCWH67) | 0.351 |
| PERMANOVA(WCWH100 vs WCWH33) | 0.251 |
| PERMANOVA(WCWH100 vs WCWH0) | 0.528 |
| PERMANOVA(WCWH67 vs WCWH33) | 0.052 |
| PERMANOVA(WCWH67 vs WCWH0) | 0.713 |
| PERMANOVA(WCWH33 vs WCWH0) | 0.256 |

^1^ WCWH0=0% of Leymus chinensis was replaced by whole crop wheat hay; WCWH33=33% of Leymus chinensis was replaced by whole crop wheat hay; WCWH67=67% of Leymus chinensis was replaced by whole crop wheat hay; WCWH100=100% of Leymus chinensis was replaced by whole crop wheat hay;

Supplementary Table 3. Effect of dietary treatments on the Phylum (as a percentage of the total sequences) of the ruminal bacterial community

| ID | WCWH100 | WCWH67 | WCWH33 | WCWH0 | p_value | q_value |
| --- | --- | --- | --- | --- | --- | --- |
| p__Bacteroidetes | 59.47 | 55.31 | 56.98 | 60.17 | 0.418 | 0.960 |
| p__Firmicutes | 35.02 | 37.92 | 32.51 | 35.34 | 0.574 | 0.960 |
| p__Proteobacteria | 1.89 | 1.82 | 3.22 | 0.91 | 0.595 | 0.960 |
| p__Fibrobacteres | 0.73 | 0.88 | 0.93 | 0.78 | 0.660 | 0.960 |
| p__Spirochaetae | 0.71 | 0.97 | 0.70 | 0.72 | 0.980 | 0.960 |
| p__Lentisphaerae | 0.61 | 0.56 | 0.83 | 0.59 | 0.688 | 0.960 |
| p__Tenericutes | 0.47 | 0.47 | 0.58 | 0.47 | 0.253 | 0.960 |
| p__Candidate_division_TM7 | 0.41 | 0.29 | 0.31 | 0.36 | 0.914 | 0.960 |
| p__Actinobacteria | 0.24 | 1.40 | 3.47 | 0.31 | 0.123 | 0.960 |
| p__Candidate_division_SR1 | 0.15 | 0.08 | 0.10 | 0.11 | 0.783 | 0.960 |
| p__SHA-109 | 0.08 | 0.07 | 0.04 | 0.06 | 0.808 | 0.960 |
| p__Cyanobacteria | 0.07 | 0.03 | 0.08 | 0.05 | 0.453 | 0.960 |
| p__unidentified | 0.04 | 0.02 | 0.04 | 0.02 | 0.264 | 0.960 |
| p__Elusimicrobia | 0.04 | 0.04 | 0.13 | 0.04 | 0.913 | 0.960 |
| p__Synergistetes | 0.03 | 0.05 | 0.04 | 0.03 | 0.547 | 0.960 |
| p__Chloroflexi | 0.03 | 0.06 | 0.03 | 0.03 | 0.519 | 0.960 |
| p__Verrucomicrobia | 0.01 | 0.00 | 0.00 | 0.00 | 0.515 | 0.960 |
| p__BD1-5 | 0.00 | 0.00 | 0.00 | 0.00 | 0.382 | 0.960 |
| p__Fusobacteria | 0.00 | 0.00 | 0.00 | 0.00 | 0.347 | 0.960 |
| p__Euryarchaeota | 0.00 | 0.00 | 0.00 | 0.00 | 0.876 | 0.960 |
| p__Planctomycetes | 0.00 | 0.00 | 0.01 | 0.00 | 0.788 | 0.960 |

Supplementary Table 4. Effect of dietary treatments on the Genus (as a percentage of the total sequences) of the ruminal bacterial community

| ID | WCWH100 | WCWH67 | WCWH33 | WCWH0 | p_value | q_value |
| --- | --- | --- | --- | --- | --- | --- |
| g__Prevotella | 33.70 | 29.86 | 32.99 | 38.89 | 0.405 | 0.879 |
| g__unidentified | 32.61 | 33.92 | 31.08 | 28.43 | 0.378 | 0.866 |
| g__RC9_gut_group | 7.77 | 7.61 | 6.97 | 7.07 | 0.664 | 0.904 |
| g__Ruminococcus | 5.20 | 7.80 | 4.45 | 7.47 | 0.131 | 0.644 |
| g__Flavonifractor | 3.09 | 4.41 | 2.59 | 3.32 | 0.218 | 0.754 |
| g__Succiniclasticum | 3.06 | 2.28 | 1.58 | 1.68 | 0.087 | 0.644 |
| g__Incertae_Sedis | 1.94 | 1.49 | 1.29 | 1.72 | 0.204 | 0.722 |
| g__unidentified_rumen_bacterium_RFN43 | 1.22 | 0.87 | 2.23 | 0.99 | 0.121 | 0.817 |
| g__Butyrivibrio | 1.12 | 1.77 | 1.40 | 1.58 | 0.241 | 0.644 |
| g__Saccharofermentans | 0.98 | 0.95 | 0.77 | 0.93 | 0.588 | 0.904 |
| g__Bacillus | 0.78 | 0.22 | 0.52 | 0.36 | 0.722 | 0.855 |
| g__Fibrobacter | 0.73 | 0.88 | 0.92 | 0.77 | 0.660 | 0.904 |
| g__Treponema | 0.62 | 0.89 | 0.63 | 0.66 | 0.905 | 0.979 |
| g__Selenomonas | 0.56 | 0.57 | 0.76 | 0.60 | 0.338 | 0.904 |
| g__Acetitomaculum | 0.44 | 0.60 | 0.53 | 0.63 | 0.143 | 0.854 |
| g__Moryella | 0.44 | 0.46 | 0.27 | 0.43 | 0.197 | 0.667 |
| g__Candidatus_Saccharimonas | 0.41 | 0.29 | 0.31 | 0.36 | 0.914 | 0.304 |
| g__Ruminobacter | 0.40 | 0.37 | 0.09 | 0.11 | 0.990 | 0.914 |
| g__Marvinbryantia | 0.36 | 0.25 | 0.28 | 0.29 | 0.689 | 0.722 |
| g__Pseudobutyrivibrio | 0.32 | 0.42 | 1.61 | 0.33 | 0.525 | 0.979 |
| g__Papillibacter | 0.31 | 0.29 | 0.28 | 0.34 | 0.821 | 0.956 |
| g__Clostridium_sensu_stricto_1 | 0.31 | 0.02 | 0.03 | 0.18 | 0.989 | 0.904 |
| g__Succinivibrio | 0.30 | 0.12 | 0.11 | 0.11 | 0.273 | 0.904 |
| g__Anaerovorax | 0.27 | 0.33 | 0.24 | 0.27 | 0.674 | 0.995 |
| g__probable_genus_10 | 0.24 | 0.15 | 0.25 | 0.20 | 0.114 | 0.644 |
| g__Paenibacillus | 0.22 | 0.08 | 0.18 | 0.11 | 0.528 | 0.644 |
| g__Bacteroides | 0.17 | 0.18 | 0.15 | 0.14 | 0.546 | 0.680 |
| g__Anaeroplasma | 0.17 | 0.20 | 0.28 | 0.20 | 0.102 | 0.822 |
| g__Mogibacterium | 0.15 | 0.15 | 0.13 | 0.14 | 0.737 | 0.904 |
| g__Victivallis | 0.14 | 0.11 | 0.21 | 0.12 | 0.656 | 0.904 |
| g__Anaerotruncus | 0.13 | 0.14 | 0.09 | 0.11 | 0.407 | 0.904 |
| g__Intestinimonas | 0.10 | 0.11 | 0.06 | 0.08 | 0.102 | 0.914 |
| g__U29-B03 | 0.10 | 0.05 | 0.05 | 0.06 | 0.950 | 0.995 |
| g__Enterococcus | 0.10 | 0.03 | 0.07 | 0.04 | 0.591 | 0.879 |
| g__Blautia | 0.10 | 0.09 | 0.09 | 0.12 | 0.127 | 0.644 |
| g__Desulfovibrio | 0.09 | 0.11 | 0.08 | 0.09 | 0.318 | 0.828 |
| g__Lactococcus | 0.09 | 0.03 | 0.04 | 0.03 | 0.695 | 0.644 |
| g__Alkaliphilus | 0.08 | 0.03 | 0.07 | 0.03 | 0.597 | 0.979 |
| g__Atopobium | 0.07 | 0.46 | 0.17 | 0.11 | 0.164 | 0.975 |
| g__Anaerovibrio | 0.07 | 0.05 | 0.07 | 0.05 | 0.695 | 0.828 |
| g__Oribacterium | 0.07 | 0.06 | 0.07 | 0.07 | 0.884 | 0.904 |
| g__Schwartzia | 0.06 | 0.03 | 0.04 | 0.02 | 0.249 | 0.904 |
| g__Candidatus_Hepatincola | 0.06 | 0.02 | 0.02 | 0.03 | 0.380 | 0.904 |
| g__Alloprevotella | 0.04 | 0.06 | 0.04 | 0.07 | 0.505 | 0.904 |
| g__Bifidobacterium | 0.04 | 0.31 | 3.10 | 0.11 | 0.350 | 0.904 |
| g__Christensenella | 0.04 | 0.05 | 0.04 | 0.04 | 0.937 | 0.904 |
| g__Thalassospira | 0.04 | 0.02 | 0.03 | 0.02 | 0.564 | 0.979 |
| g__Coprococcus | 0.04 | 0.06 | 0.03 | 0.04 | 0.074 | 0.644 |
| g__Megasphaera | 0.03 | 0.04 | 0.00 | 0.00 | 0.158 | 0.818 |
| g__M2PT2-76_termite_group | 0.03 | 0.02 | 0.02 | 0.01 | 0.154 | 0.914 |
| g__Phocaeicola | 0.03 | 0.03 | 0.01 | 0.03 | 0.148 | 0.979 |
| g__Elusimicrobium | 0.03 | 0.02 | 0.11 | 0.03 | 0.653 | 0.866 |
| g__Succinimonas | 0.03 | 0.04 | 0.01 | 0.03 | 0.864 | 0.904 |
| g__unidentified_rumen_bacterium_RFP12 | 0.03 | 0.02 | 0.04 | 0.02 | 0.179 | 0.975 |
| g__Pyramidobacter | 0.03 | 0.04 | 0.03 | 0.03 | 0.942 | 0.669 |
| g__Enterorhabdus | 0.03 | 0.04 | 0.03 | 0.03 | 0.455 | 0.904 |
| g__Syntrophococcus | 0.03 | 0.03 | 0.04 | 0.03 | 0.744 | 0.683 |
| g__Howardella | 0.02 | 0.02 | 0.03 | 0.02 | 0.499 | 0.904 |
| g__Enterobacter | 0.02 | 0.00 | 0.01 | 0.01 | 0.121 | 0.675 |
| g__Spirochaeta | 0.02 | 0.02 | 0.02 | 0.01 | 0.638 | 0.904 |
| g__Pantoea | 0.02 | 0.01 | 0.01 | 0.00 | 0.134 | 0.675 |
| g__Deltaproteobacteria_bacterium_canine_oral_taxon_266 | 0.01 | 0.02 | 0.01 | 0.01 | 0.665 | 0.914 |
| g__Staphylococcus | 0.01^a^ | 0.00^b^ | 0.00^b^ | 0.00^b^ | 0.001 | 0.904 |
| g__Streptococcus | 0.01 | 0.02 | 0.02 | 0.01 | 0.745 | 0.644 |
| g__Roseburia | 0.01 | 0.01 | 0.01 | 0.02 | 0.932 | 0.979 |
| g__Shuttleworthia | 0.01 | 0.02 | 0.21 | 0.02 | 0.313 | 0.904 |
| g__Anaerosporobacter | 0.01 | 0.01 | 0.00 | 0.01 | 0.995 | 0.644 |
| g__unidentified_rumen_bacterium_RF9 | 0.01 | 0.01 | 0.01 | 0.01 | 0.953 | 0.644 |
| g__Pseudomonas | 0.01 | 0.00 | 0.01 | 0.00 | 0.626 | 0.304 |
| g__Saccharopolyspora | 0.01^a^ | 0.00^ab^ | 0.00^b^ | 0.00^b^ | 0.005 | 0.995 |
| g__Psychrobacter | 0.01 | 0.00 | 0.00 | 0.00 | 0.799 | 0.979 |
| g__unidentified_rumen_bacterium_RFN54 | 0.01 | 0.00 | 0.01 | 0.01 | 0.457 | 0.904 |
| g__Oryza_sativa_Japonica_Group | 0.01 | 0.01 | 0.01 | 0.01 | 0.623 | 0.855 |
| g__Solobacterium | 0.01 | 0.01 | 0.01 | 0.01 | 0.566 | 0.904 |
| g__Clostridium_sensu_stricto_18 | 0.01 | 0.00 | 0.00 | 0.00 | 0.663 | 0.975 |
| g__Laceyella | 0.01^a^ | 0.00^ab^ | 0.00^b^ | 0.00^b^ | 0.006 | 0.979 |
| g__Desulfobulbus | 0.01 | 0.01 | 0.01 | 0.00 | 0.912 | 0.862 |
| g__Carnobacterium | 0.01 | 0.00 | 0.00 | 0.00 | 0.383 | 0.904 |
| g__Acinetobacter | 0.01 | 0.00 | 0.00 | 0.00 | 0.161 | 0.557 |
| g__Lactobacillus | 0.00 | 0.00 | 0.02 | 0.00 | 0.446 | 0.866 |
| g__Brevibacillus | 0.01 | 0.00 | 0.00 | 0.00 | 0.790 | 0.937 |
| g__Turicibacter | 0.00 | 0.00 | 0.01 | 0.01 | 0.346 | 0.644 |
| g__unidentified_rumen_bacterium_RF3 | 0.00 | 0.01 | 0.00 | 0.01 | 0.883 | 0.818 |
| g__Oscillospira | 0.00 | 0.00 | 0.04 | 0.01 | 0.134 | 0.904 |
| g__Oscillibacter | 0.00 | 0.00 | 0.00 | 0.00 | 0.512 | 0.866 |
| g__Planomicrobium | 0.00 | 0.00 | 0.00 | 0.00 | 0.693 | 0.937 |
| g__Anaerofustis | 0.00 | 0.01 | 0.00 | 0.00 | 0.638 | 0.132 |
| g__Leuconostoc | 0.00 | 0.00 | 0.00 | 0.00 | 0.066 | 0.904 |
| g__Streptomyces | 0.00^a^ | 0.00^ab^ | 0.00^ab^ | 0.00^b^ | 0.018 | 0.904 |
| g__Sporobacter | 0.00 | 0.00 | 0.00 | 0.00 | 0.743 | 0.975 |
| g__unidentified_rumen_bacterium_RFN82 | 0.00 | 0.00 | 0.00 | 0.00 | 0.865 | 0.914 |
| g__Arthrobacter | 0.00 | 0.00 | 0.00 | 0.00 | 0.084 | 0.132 |
| g__Geobacillus | 0.00 | 0.00 | 0.00 | 0.00 | 0.749 | 0.904 |
| g__Brochothrix | 0.00 | 0.00 | 0.00 | 0.00 | 0.417 | 0.904 |
| g__SP3-e08 | 0.00 | 0.00 | 0.01 | 0.01 | 0.359 | 0.904 |
| g__Thermoactinomyces | 0.00 | 0.00 | 0.00 | 0.00 | 0.169 | 0.907 |
| g__Syntrophomonas | 0.00 | 0.00 | 0.00 | 0.00 | 0.178 | 0.904 |
| g__Escherichia-Shigella | 0.00 | 0.00 | 0.00 | 0.00 | 0.575 | 0.904 |
| g__Synergistes | 0.00^a^ | 0.00^ab^ | 0.00^b^ | 0.00^b^ | 0.027 | 0.995 |
| g__Curtobacterium | 0.00 | 0.00 | 0.00 | 0.00 | 0.431 | 0.682 |
| g__Corynebacterium | 0.00 | 0.00 | 0.00 | 0.00 | 0.130 | 0.360 |
| g__Ralstonia | 0.00 | 0.00 | 0.00 | 0.00 | 0.268 | 0.644 |
| g__Fretibacterium | 0.00 | 0.01 | 0.01 | 0.00 | 0.367 | 0.683 |
| g__Hydrogenoanaerobacterium | 0.00 | 0.00 | 0.00 | 0.00 | 0.706 | 0.377 |
| g__Rhizobium | 0.00 | 0.00 | 0.00 | 0.00 | 0.203 | 0.975 |
| g__Olsenella | 0.00 | 0.00 | 0.01 | 0.00 | 0.562 | 0.822 |
| g__Candidatus_Captivus | 0.00 | 0.01 | 0.00 | 0.00 | 0.126 | 0.557 |
| g__Clostridium_sensu_stricto_3 | 0.00 | 0.00 | 0.00 | 0.00 | 0.516 | 0.914 |
| g__Clostridium_sensu_stricto_13 | 0.00 | 0.00 | 0.00 | 0.00 | 0.791 | 0.722 |
| g__possible_genus_Sk003-Sk004 | 0.00 | 0.00 | 0.00 | 0.00 | 0.905 | 0.937 |
| g__Shimazuella | 0.00 | 0.00 | 0.00 | 0.00 | 0.125 | 0.979 |
| g__Quinella | 0.00^b^ | 0.01^b^ | 0.01^ab^ | 0.02^a^ | 0.019 | 0.904 |
| g__Aegilops_tauschii | 0.00 | 0.00 | 0.00 | 0.00 | 0.979 | 0.644 |
| g__Actinopolyspora | 0.00 | 0.00 | 0.00 | 0.00 | 0.081 | 0.644 |
| g__Saccharomonospora | 0.00 | 0.00 | 0.00 | 0.00 | 0.097 | 0.937 |
| g__Brevundimonas | 0.00 | 0.00 | 0.00 | 0.00 | 0.258 | 0.887 |
| g__Noviherbaspirillum | 0.00 | 0.00 | 0.00 | 0.00 | 0.880 | 0.644 |
| g__Nocardiopsis | 0.00 | 0.00 | 0.00 | 0.00 | 0.096 | 0.644 |
| g__Comamonas | 0.00 | 0.00 | 0.00 | 0.00 | 0.698 | 0.904 |
| g__Methanobrevibacter | 0.00 | 0.00 | 0.00 | 0.00 | 0.876 | 0.822 |
| g__Collinsella | 0.00 | 0.00 | 0.00 | 0.00 | 0.482 | 0.644 |
| g__dgA-11_gut_group | 0.00 | 0.00 | 0.00 | 0.00 | 0.312 | 0.644 |
| g__hoa5-07d05_gut_group | 0.00 | 0.00 | 0.01 | 0.00 | 0.252 | 0.904 |
| g__Prauserella | 0.00 | 0.00 | 0.00 | 0.00 | 0.289 | 0.822 |
| g__Stenotrophomonas | 0.00 | 0.00 | 0.00 | 0.00 | 0.862 | 0.904 |
| g__Peptostreptococcus | 0.00 | 0.00 | 0.00 | 0.00 | 0.088 | 0.937 |
| g__Weissella | 0.00 | 0.00 | 0.00 | 0.00 | 0.293 | 0.975 |
| g__Sphingobacterium | 0.00 | 0.00 | 0.00 | 0.00 | 0.392 | 0.979 |
| g__Paludibacter | 0.00 | 0.00 | 0.00 | 0.00 | 0.541 | 0.828 |
| g__Sphingomonas | 0.00 | 0.00 | 0.00 | 0.00 | 0.586 | 0.818 |
| g__Pseudoramibacter | 0.00 | 0.00 | 0.00 | 0.00 | 0.490 | 0.828 |
| g__Ochrobactrum | 0.00 | 0.00 | 0.00 | 0.00 | 0.938 | 0.975 |
| g__Denitrobacterium | 0.00 | 0.00 | 0.00 | 0.00 | 0.322 | 0.904 |
| g__Advenella | 0.00 | 0.00 | 0.00 | 0.00 | 0.293 | 0.644 |
| g__Trichococcus | 0.00 | 0.00 | 0.00 | 0.00 | 0.105 | 0.904 |
| g__Rhodococcus | 0.00 | 0.00 | 0.00 | 0.00 | 0.105 | 0.904 |
| g__Mitsuokella | 0.00 | 0.00 | 0.00 | 0.00 | 0.562 | 0.822 |
| g__Enhydrobacter | 0.00 | 0.00 | 0.00 | 0.00 | 0.642 | 0.904 |
| g__Sutterella | 0.00 | 0.00 | 0.00 | 0.00 | 0.121 | 0.822 |
| g__Atopostipes | 0.00 | 0.00 | 0.00 | 0.00 | 0.562 | 0.644 |
| g__Methylobacterium | 0.00 | 0.00 | 0.00 | 0.00 | 0.799 | 0.904 |
| g__Allisonella | 0.00 | 0.00 | 0.00 | 0.00 | 0.788 | 0.822 |
| g__vadinBC27_wastewater-sludge_group | 0.00 | 0.00 | 0.00 | 0.00 | 0.293 | 0.871 |
| g__Blvii28_wastewater-sludge_group | 0.00 | 0.00 | 0.00 | 0.00 | 0.298 | 0.904 |
| g__Anaerobiospirillum | 0.00 | 0.00 | 0.00 | 0.00 | 0.561 | 0.644 |
| g__Dethiosulfatibacter | 0.00 | 0.00 | 0.00 | 0.00 | 0.562 | 0.644 |
| g__Pelagibacterium | 0.00 | 0.00 | 0.00 | 0.00 | 0.562 | 0.904 |
| g__Tannerella | 0.00 | 0.00 | 0.00 | 0.00 | 0.562 | 0.904 |
| g__Eubacterium | 0.00 | 0.00 | 0.00 | 0.00 | 0.562 | 0.904 |
